# Vehicle Localization in 3D World Coordinates Using Single Camera at Traffic Intersection

**DOI:** 10.3390/s23073661

**Published:** 2023-03-31

**Authors:** Shenglin Li, Hwan-Sik Yoon

**Affiliations:** Department of Mechanical Engineering, The University of Alabama, Tuscaloosa, AL 35487, USA; sli90@crimson.ua.edu

**Keywords:** vehicle localization, computer vision, object detection, machine learning, camera calibration

## Abstract

Optimizing traffic control systems at traffic intersections can reduce the network-wide fuel consumption, as well as emissions of conventional fuel-powered vehicles. While traffic signals have been controlled based on predetermined schedules, various adaptive signal control systems have recently been developed using advanced sensors such as cameras, radars, and LiDARs. Among these sensors, cameras can provide a cost-effective way to determine the number, location, type, and speed of the vehicles for better-informed decision-making at traffic intersections. In this research, a new approach for accurately determining vehicle locations near traffic intersections using a single camera is presented. For that purpose, a well-known object detection algorithm called YOLO is used to determine vehicle locations in video images captured by a traffic camera. YOLO draws a bounding box around each detected vehicle, and the vehicle location in the image coordinates is converted to the world coordinates using camera calibration data. During this process, a significant error between the center of a vehicle’s bounding box and the real center of the vehicle in the world coordinates is generated due to the angled view of the vehicles by a camera installed on a traffic light pole. As a means of mitigating this vehicle localization error, two different types of regression models are trained and applied to the centers of the bounding boxes of the camera-detected vehicles. The accuracy of the proposed approach is validated using both static camera images and live-streamed traffic video. Based on the improved vehicle localization, it is expected that more accurate traffic signal control can be made to improve the overall network-wide energy efficiency and traffic flow at traffic intersections.

## 1. Introduction

Traffic congestion affects not only fuel consumption but also the emissions of conventional fuel-powered vehicles in a traffic network. Due to this fact, a great deal of effort has been made to improve the performance of the traffic signal control system at traffic intersections. Traditionally, traffic signals have been controlled based on fixed or varying schedules called the signal phase and timing (SPaT) [1]. Thanks to improving computer hardware and the increasing amount of data in the transportation domain, adaptive control systems were developed for traffic signals, such as memetic algorithm for traffic signal optimization [2] and traffic signal controller based on fuzzy logic [3]. In contrast to the traditional schedule-based traffic signal control systems, these systems change the traffic signal based on the current traffic condition detected by sensors, such as induction-loop. Recently, advanced sensors, including cameras, radars, and LiDARs, have been considered for traffic monitoring to provide more detailed information such as the number, location, and speed of the vehicles for better-informed decision-making at traffic intersections. Examples include the Sydney coordinated adaptive traffic (SCATS) [4], split cycle time and the offset optimization technique (SCOOT) [5], real-time hierarchical optimizing distributed effective system (RHODES) [6], experiences with adaptive signal control in Germany [7], and the smart traffic congestion control system [8]. These adaptive control systems optimize the SPaT in real time to minimize delay and stops of the vehicles at each intersection. Among those advanced sensors, camera-based systems can provide detailed information on traffic conditions in a cost-effective manner due to the relatively inexpensive camera modules.

Camera-based object detection is widely used in many fields, including autonomous driving, transportation, robotics, and even medicine. Based on images captured by the camera hardware, various features of objects can be identified using an object detection algorithm. One of the well-known object detection algorithms is you only look once (YOLO) [9,10,11,12]. YOLO uses a unique convolutional neural network, darknet framework, to detect objects as a regression problem and provides class probabilities for the detected objects in a single run. Therefore, detected objects are shown in a rectangular box to inform the type, size, and location of the objects. Compared with other algorithms such as single shot multi-box detector (SSD) [13], Faster-RCNN [14], Mask-RCNN [15], and CenterNet [16], YOLO features high accuracy and fast computational speed. When applied to transportation, YOLO can draw a bounding box for every detected vehicle in an image allowing estimation of the position of multiple vehicles in the scene.

Through coordinate transformation using camera calibration data [17], a vehicle’s position obtained by YOLO in the two-dimensional (2D) image coordinates can be converted into three-dimensional (3D) world coordinates. Real-time counting of vehicles in each traffic lane based on the vehicles’ position can be used by the traffic signal control system to prioritize multiple approaching lanes and determine which lane will reach the next green light based on the number of vehicles and their total waiting time. However, even though the bounding box of a vehicle drawn by YOLO provides a vehicle’s position with a relatively high degree of accuracy in the image coordinates, there is a geometrical issue that causes a significant error between the center of a vehicle’s bounding box and the real center of the vehicle in the world coordinates. This error caused by the camera view angles leads to an inaccurate vehicle localization and count in each lane. Therefore, to reduce the vehicle localization error, a new method to correct the center of vehicles detected by YOLO has been developed and is presented in this paper.

This paper is organized as follows. After related works are presented, the camera calibration method is presented to calculate the transformation matrix between the 2D image coordinates and the 3D world coordinates. Then, a regression model-based approach is presented to correct the geometrical error of the vehicle center location. Finally, validation results are presented for single vehicles in still images and multiple vehicles in live-streamed videos.

## 2. Related Works

Various methods have been developed to accurately locate vehicles at a traffic intersection by using advanced sensors, such as LiDARs, together with state-of-the-art machine learning algorithms. For example, a method for road sign detection using a LiDAR has been reported to improve vehicle localization in an HD map [18]. Another method utilizes points clouds from LiDARs and an Euler region proposal algorithm to generate region proposals for each vehicle in the point clouds [19]. These methods can improve detection accuracy, which in turn can have significant applications in autonomous driving and other advanced driver assistance systems. Despite their wide applications, LiDARs do have some drawbacks, including their high cost and maintenance requirements, as well as the challenge of accurately classifying certain types of vehicles, such as motorcycles, sedans, and trucks.

Other methods utilize cameras as sensors with deep learning algorithms. For example, a study used several deep learning methods and a novel data association algorithm to estimate and monitor traffic flow on a highway in real time [20]. By providing accurate and reliable traffic flow estimation, the system can aid in effective traffic management and help reduce congestion on highways. However, the use of the complex system requires high computational power, which may limit its application in certain scenarios. Another study used a combination of stereo vision and deep learning to reconstruct accurate vehicle locations in a traffic scene [21]. The system utilizes multiple cameras to capture a video of a traffic scene, then the video frames are processed by a deep neural network to construct 3D bounding boxes around the detected vehicles. The system determines vehicle location and speed by using the 3D bounding boxes and camera calibration data. It also classifies the vehicle types based on the size of the 3D bounding box. Their results showed that the average vehicle localization error was 1.81 m using a differential GPS as a reference and 1.68 m using a drone as a reference. Additionally, other studies employed computer vision algorithms to estimate and monitor traffic flow using multiple cameras, which could improve traffic safety and efficiency in a cost-effective manner [22,23].

In this paper, a new simple and effective approach is presented to reduce the localization error of the vehicle detected by YOLO for traffic control applications. The proposed method uses two different types of regression models to estimate the distance between the center of a vehicle’s bounding box and the center of the vehicle’s projection on the road. This estimated distance is then used to correct the vehicle localization error. In comparison to other previous methods, this new regression model-based approach can correct the vehicle localization error faster with an acceptable accuracy using a single camera.

**Figure 1:**
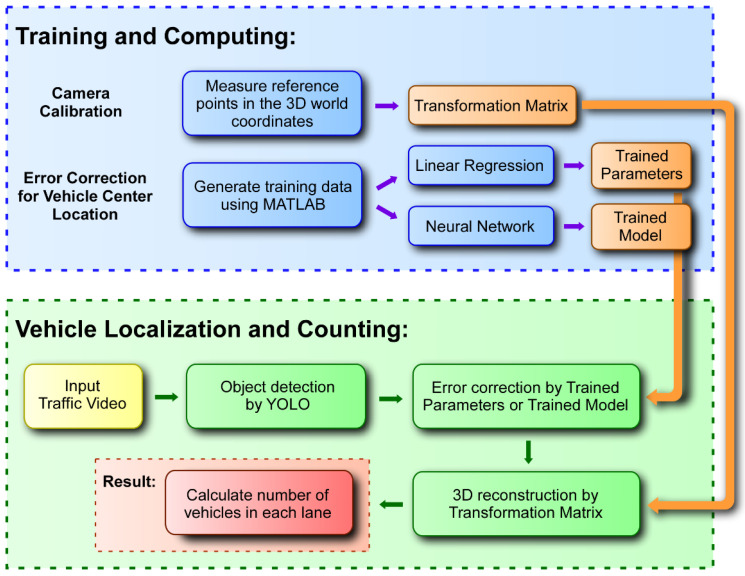
Overall workflow of the proposed vehicle localization method.

The overall workflow of the proposed vehicle localization method is shown in Figure 1. In the figure, it is shown that the camera calibration is conducted, and error correction models are developed in the training and computing phase. The resulting models are then applied to the input traffic video in the vehicle localization and counting phase to evaluate the effectiveness of the proposed method.

## 3. Vehicle Detection and Localization

### 3.1. Vehicle Detection Algorithm

For the vehicle detection using a camera installed on a traffic light pole, an image-based object detection algorithm is utilized. Object detection algorithms based on a convolutional neural network such as YOLO are known for their high accuracy and real-time performance. The fourth version of YOLO, YOLOv4, uses darknet convolutional neural networks as the backbone, spatial pyramid pooling (SPP) [24] and path aggregation network (PANet) [25] as the neck, and YOLOv3 as the head. Many features are used to improve the accuracy while maintaining real-time performance in YOLOv4. For the current transportation application, it is only necessary to detect motorcycles, cars, and trucks, and thus only those categories are turned on among various types of objects that YOLOv4 can detect. To determine the physical locations of the detected vehicles, 2D coordinates of the center of the bounding box of each vehicle in the image should be transformed into the corresponding 3D world coordinates. This coordinate transformation can be performed by using matrices obtained from the camera calibration.

### 3.2. Camera Calibration

**Figure 2:**
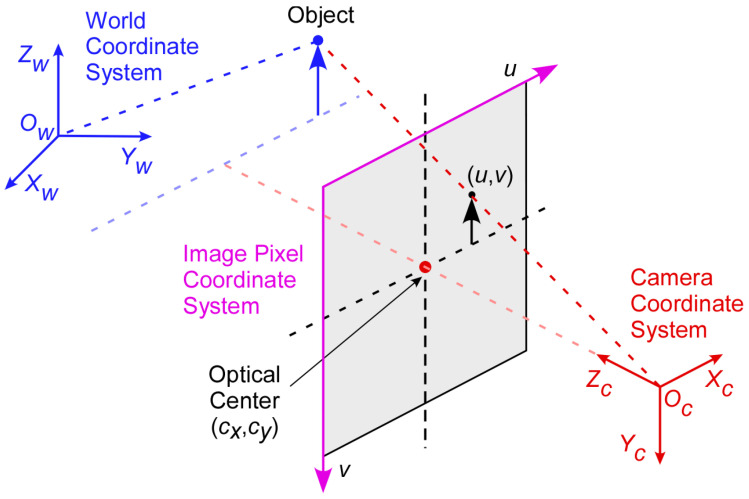
Three coordinate systems used in the camera calibration.

Camera calibration is the process of determining coordinate transformation matrices. For this purpose, three coordinate systems are used: 2D image pixel, 3D camera, and 3D world coordinate systems, as shown in Figure 2. For the origin of the 3D world coordinate system, the location of the camera fixed on the traffic light pole is used. Based on a previous work [26,27], the coordinate transformation matrix, P, is defined as follows:

(1) P=Intrinsic Matrix×Extrinsic Matrix,

where the Intrinsic Matrix is used to transform the 3D camera coordinate system to the 2D image coordinate system and the Extrinsic Matrix transforms the 3D world coordinate system to the 3D camera coordinate system. Through the camera calibration, a mathematical relationship between the 2D image pixel coordinates and its corresponding 3D world coordinates of detected vehicles can be determined.

#### 3.2.1. Transformation between Three Coordinates

The 3D world coordinates can be transformed into the 3D camera coordinates using the extrinsic matrix $\left[R_{o}|T\right]$ based on the following relationship:

(2) $$\left[\begin{array}{c} x_{c}\\ y_{c}\\ z_{c} \end{array}\right]=\left[R_{o}|T\right]\left[\begin{array}{c} x_{w}\\ y_{w}\\ z_{w}\\ 1 \end{array}\right],$$

where $\left(x_{c},\;y_{c},\;z_{c}\right)$ and $\left(x_{w},\;y_{w},\;z_{w}\right)$ represent the camera coordinates and the world coordinates, respectively. In the extrinsic matrix, $R_{o}$ is a 3 × 3 rotation matrix, and T is a 3 × 1 translation vector. In this application, the height coordinate, $z_{w}$, in the world coordinates is set to 0, assuming that the road is level and all vehicles have the same vertical coordinates. However, for the completeness of the equation derivation, the variable, $z_{w}$, is carried over until the last stage where 0 is assigned to the variable.

The 3D camera coordinates can be transformed into the 2D image coordinates using an intrinsic matrix, K defined by:

(3) $$K=\left[\begin{array}{ccc} f & 0 & c_{x}\\ 0 & f & c_{y}\\ 0 & 0 & 1 \end{array}\right],$$

where f is the focal length, and $\left(c_{x},\;c_{y}\right)$ are the coordinates of the optical center of the camera, which may not coincide with the center of the image coordinate system. The relationship between the 2D image coordinates and the 3D camera coordinates can be represented by:

(4) $$s\left[\begin{array}{c} u\\ v\\ 1 \end{array}\right]=K\left[\begin{array}{c} x_{c}\\ y_{c}\\ z_{c} \end{array}\right],$$

where $\left(u,v\right)$ is a pixel location in the image pixel coordinate system and s is a scaling factor.

Finally, the transformation between the 2D image coordinates and the 3D world coordinates can be obtained by combining Equations (2) and (4) as:

(5) $$s\left[\begin{array}{c} u\\ v\\ 1 \end{array}\right]=P\left[\begin{array}{c} x_{w}\\ y_{w}\\ z_{w}\\ 1 \end{array}\right],$$

where the 3 × 4 transformation matrix P is defined by:

(6) $$P=K\times \left[R_{o}|T\right].$$

With an expansion of the matrix, P, Equation (5) can be rewritten as:

(7) $$s\;\left[\begin{array}{c} u\\ v\\ 1 \end{array}\right]=\left[\begin{array}{cccc} p_{1} & p_{2} & p_{3} & p_{4}\\ p_{5} & p_{6} & p_{7} & p_{8}\\ p_{9} & p_{10} & p_{11} & p_{12} \end{array}\right]\left[\begin{array}{c} x_{w}\\ y_{w}\\ z_{w}\\ 1 \end{array}\right]$$

Expanding Equation (7) produces the following three equations:

(8) $$\left\{\begin{array}{c} u=\left(p_{1}x_{w}+p_{2}y_{w}+p_{3}z_{w}+p_{4}\right)/s\\ v=\left(p_{5}x_{w}+p_{6}y_{w}+p_{7}z_{w}+p_{8}\right)/s\\ s=p_{9}x_{w}+p_{10}y_{w}+p_{11}z_{w}+p_{12} \end{array}\right.$$

If there are N different points in the 3D world coordinates with their corresponding 2D projections, moving all terms to the left-hand side for the 3N different equations will lead to the following homogeneous linear system [28]:

(9) $$\left[\begin{array}{cccccccccccc} x_{w}^{\left(1\right)} & y_{w}^{\left(1\right)} & z_{w}^{\left(1\right)} & 1 & 0 & 0 & 0 & 0 & -u^{\left(1\right)}x_{w}^{\left(1\right)} & -u^{\left(1\right)}y_{w}^{\left(1\right)} & -u^{\left(1\right)}z_{w}^{\left(1\right)} & -u^{\left(1\right)}\\ 0 & 0 & 0 & 0 & x_{w}^{\left(1\right)} & y_{w}^{\left(1\right)} & z_{w}^{\left(1\right)} & 1 & -v^{\left(1\right)}x_{w}^{\left(1\right)} & -v^{\left(1\right)}y_{w}^{\left(1\right)} & -v^{\left(1\right)}z_{w}^{\left(1\right)} & -v^{\left(1\right)}\\ \vdots & \vdots & \vdots & \vdots & \vdots & \vdots & \vdots & \vdots & \vdots & \vdots & \vdots & \vdots \\ x_{w}^{\left(N\right)} & y_{w}^{\left(N\right)} & z_{w}^{\left(N\right)} & 1 & 0 & 0 & 0 & 0 & -u^{\left(N\right)}x_{w}^{\left(N\right)} & -u^{\left(N\right)}y_{w}^{\left(N\right)} & -u^{\left(N\right)}z_{w}^{\left(N\right)} & -u^{\left(N\right)}\\ 0 & 0 & 0 & 0 & x_{w}^{\left(N\right)} & y_{w}^{\left(N\right)} & z_{w}^{\left(N\right)} & 1 & -v^{\left(N\right)}x_{w}^{\left(N\right)} & -v^{\left(N\right)}y_{w}^{\left(N\right)} & -v^{\left(N\right)}z_{w}^{\left(N\right)} & -v^{\left(N\right)} \end{array}\right]\;\left[\begin{array}{c} p_{1}\\ p_{2}\\ p_{3}\\ p_{4}\\ p_{5}\\ p_{6}\\ p_{7}\\ p_{8}\\ p_{9}\\ p_{10}\\ p_{11}\\ p_{12} \end{array}\right]=0$$

The homogeneous linear equation can be solved by using the singular value decomposition (SVD) method [29]. When tested with different numbers of data points in a small-scale setup, the homogeneous equation produced the best result with sixteen data points. Therefore, sixteen points are used in this research to determine the coordinate transformation matrix.

#### 3.2.2. QR Decomposition

It is challenging to measure physical locations of the data points on the road from the origin of the world coordinate system fixed on a traffic pole. The solution is to select a new coordinate origin from which it is relatively easier to measure physical locations of the data points, and then translate the coordinate origin from the newly chosen point to the traffic pole using QR decomposition [30]. A combination of the camera calibration result based on the new origin of the world coordinates, and the distance between the new origin and the traffic pole produces a result that is roughly equal to the one based on the traffic pole as the origin. The QR decomposition-based method provides a convenient and inexpensive way to measure the world coordinates of the data points with an acceptable error.

The QR factorization is a decomposition of a matrix into an orthogonal matrix Q and an upper triangular matrix R. First, the transformation matrix P is rewritten as follows:

(10) $$P=K\left[R_{o}|T\right]=\left[KR_{o}|KT\right]$$

The translation matrix, T, can also be represented by:

(11) $$T=-R_{o}C,$$

where C is a 3 × 1 vector representing the camera’s position in the world coordinates. By introducing a 3 × 3 matrix M defined as:

(12) $$M=KR_{o},$$

the following equation can be obtained:

(13) $$P=\left[M|-MC\right]$$

Finally, from Equations (11) and (12), the following relationships can be obtained:

(14) $$R_{o}=K^{-1}M$$

(15) $$T=-R_{o}C=-K^{-1}MC$$

In this way, the intrinsic matrix K, rotation matrix $R_{o}$, and translation matrix T can all be obtained. The translation matrix, T, represents the distance between the new coordinate origin and the traffic pole. Each camera has its own associated transformation matrix, which does not change as long as the camera’s view angles are fixed. Therefore, camera calibration needs to be performed only once for each camera.

## 4. Error Correction for Vehicle Center Location

**Figure 3:**
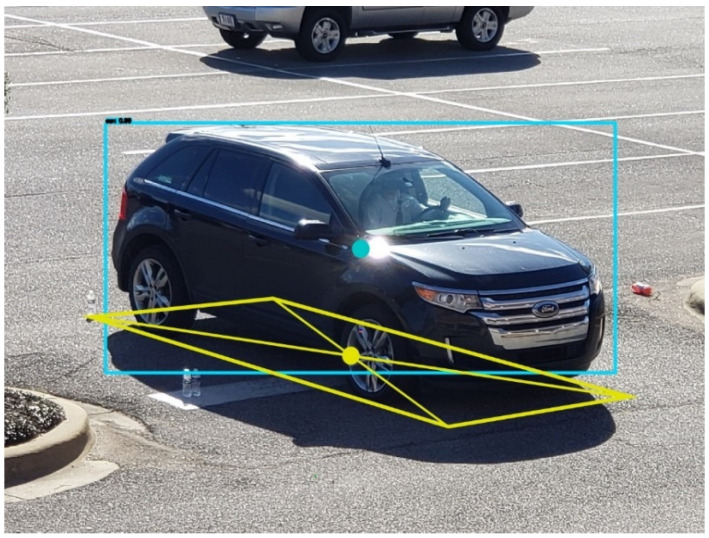
Error between the center of the bounding box and the true vehicle center.

There is an inherent geometrical error in obtaining the vehicle center location in the world coordinates from the corresponding 2D image coordinates that YOLO provides. When YOLO detects a vehicle, it draws a bounding box around the vehicle and the center of the bounding box is regarded as the center of the vehicle. However, due to the angled view of vehicles by the traffic camera, the center of the bounding box does not match with the true center of the vehicle on the road. This error can be explained using the vehicle shown in Figure 3, where the center of the bounding box is shown as a blue dot and the true center of the vehicle is shown as a yellow dot. Note that the yellow dot is at the intersection of the diagonals of a parallelogram obtained by projecting the vehicle onto the ground.

Since the error in locating the vehicle center depends on the vehicle size and the camera view angles, it is possible to develop a regression model that relates the error to the vehicle size and the camera view angles. In this research, two different types of regression models are utilized: a linear regression function and a nonlinear neural network.

### 4.1. Error in Vehicle Center Localization

**Figure 4:**
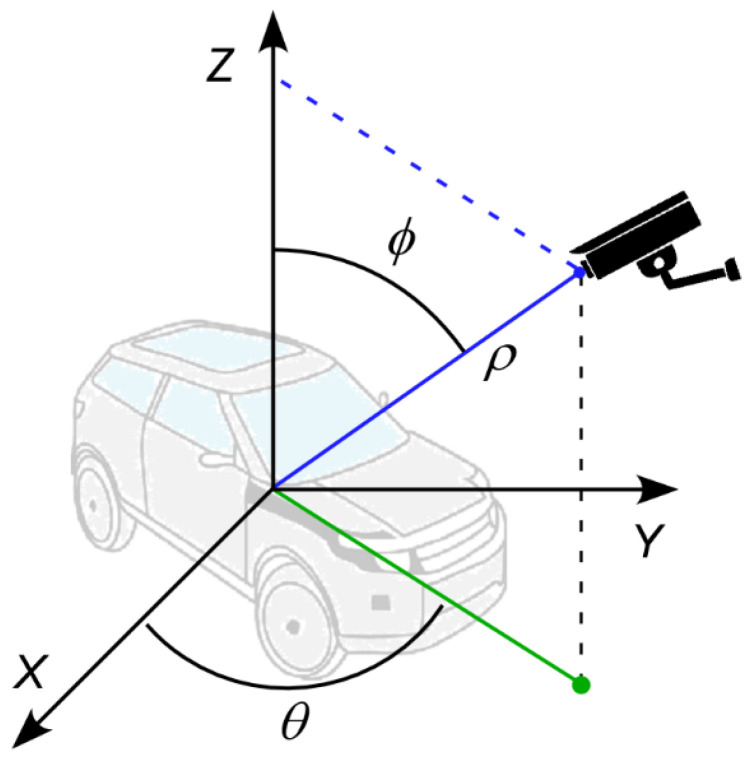
Camera view of a vehicle in the spherical coordinate system.

Vehicles at different locations seen by a fixed camera appear in different sizes and orientations. In the spherical coordinate system, a vehicle can be represented by two observation angles, θ and 𝜙, and distance, ρ, as shown in Figure 4 [31]. The error of the vehicle center location caused by the varying view angles and distance can be represented and thus corrected by these variables.

For the training of a regression model, the input features of the training dataset may include the height and width of the vehicle bounding box, distance between the vehicle and the camera, and two observation angles from the camera. While the distance between the vehicle and the camera is difficult to measure in a real situation, the distance is related to the size of the bounding box. More specifically, the size of the bounding box decreases as the distance between the vehicle and the camera increases. Therefore, the distance, ρ, may be ignored as a dependent variable. Additionally, the azimuth angle, θ, is related to the width of the bounding box without noticeably changing the height of the bounding box. Therefore, the azimuth angle, θ, can be ignored as a dependent variable as well. As a result, only the vehicle size represented by the bounding box height and width, and the elevation angle, 𝜙, can be meaningful input features to the regression model.

### 4.2. Regression Models for Vehicle Center Error Correction

**Figure 5:**
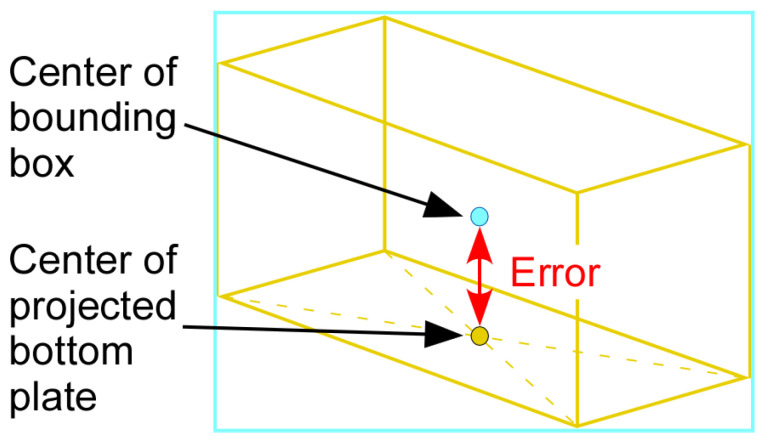
Difference between the center of a bounding box and the center of the bottom plate of a rectangular prism.

To obtain the training dataset, various images of 3D rectangular prisms are generated by using MATLAB and then 2D bounding boxes are drawn around the 3D rectangular prisms as shown in Figure 5. In the figure, the distance between the center of the bounding box and the projection of the center of the rectangular prism onto the bottom plate is used as the output of the regression model.

For the regression model to cover a large number of vehicles of different sizes, the prediction output needs to be normalized with the size of the bounding box before model training. Thus, the prediction output of the regression model is defined by:

(16) $$prediction=\frac{1}{2}\times \left(\frac{error}{bounding\;box\;height}+\frac{error}{bounding\;box\;width}\right),$$

where prediction is a normalized value between 0 and 1, and error is the vertical distance between the blue and yellow dots in the rectangular prism image shown in Figure 5. The error can be calculated from Equation (16) as:

(17) $$error=2\left(\frac{\;bounding\;box\;height\times bounding\;box\;width}{bounding\;box\;height+bounding\;box\;width}\right)\times prediction$$

For the linear regression model, it would appear that the elevation angle, 𝜙, does not noticeably affect the output. Thus, only the height and width of the bounding box are used as the input features. The linear hypothesis, h, and the error function, J, can be expressed as:

(18) $$h_{a}\left(x\right)=a_{0}+a_{1}x_{1}+a_{2}x_{2}$$

(19) $$J\left(a\right)=\frac{1}{2N}\sum_{i=1}^{N}{\left(h_{a}\left(x^{\left(i\right)}\right)-y^{\left(i\right)}\right)}^{2}$$

where $a^{\prime }s$ are model parameters, $x^{\prime }s$ are input features, N is the number of examples, and y is the output error from Equation (17).

For the nonlinear neural network model, the input features include the height and width of the bounding box and the elevation angle, 𝜙. Here, the elevation angle, 𝜙, can be calculated by using the QR decomposition in the camera calibration step. A simple neural network consisting of two hidden layers is used with 10 nodes in the first hidden layer and five nodes in the second hidden layer.

## 5. Validation of Vehicle Localization Error Correction

The proposed vehicle localization error correction method is evaluated quantitatively in both static and dynamic scenarios. Additionally, the accuracy in vehicle counting is assessed using two different traffic scenarios, one with light traffic volume and the other with heavy traffic volume. The results of these evaluations demonstrate the effectiveness of the method in improving the accuracy of vehicle localization and counting.

### 5.1. Three-Dimensional Position Reconstruction

Once the vehicle location in the 2D image coordinates, $\left(u,v\right)$, has been detected by YOLO and corrected by one of the regression models, the next step is to convert the vehicle’s location to the 3D world coordinates using the transformation matrix, P. Based on Equation (7), the vehicle location in the 3D world coordinates can be obtained by:

(20) $$\begin{array}{c} x_{w}=s\left(p_{1}^{\prime }u+p_{2}^{\prime }v+p_{3}^{\prime }\right)-\left(p_{1}^{\prime }p_{4}+p_{2}^{\prime }p_{8}+p_{3}^{\prime }p_{12}\right)\\ y_{w}=s\left(p_{5}^{\prime }u+p_{6}^{\prime }v+p_{7}^{\prime }\right)-\left(p_{5}^{\prime }p_{4}+p_{6}^{\prime }p_{8}+p_{7}^{\prime }p_{12}\right)\\ z_{w}=s\left(p_{9}^{\prime }u+p_{10}^{\prime }v+p_{11}^{\prime }\right)-\left(p_{9}^{\prime }p_{4}+p_{10}^{\prime }p_{8}+p_{11}^{\prime }p_{12}\right) \end{array}$$

Assuming that the road surface is even, and the vehicle center is on the road, i.e., $z_{w}=0$, Equation (20) can be simplified as:

(21) $$\begin{array}{c} x_{w}=s\left(p_{1}^{\prime }u+p_{2}^{\prime }v+p_{3}^{\prime }\right)-\left(p_{1}^{\prime }p_{4}+p_{2}^{\prime }p_{8}+p_{3}^{\prime }p_{12}\right)\\ y_{w}=s\left(p_{5}^{\prime }u+p_{6}^{\prime }v+p_{7}^{\prime }\right)-\left(p_{5}^{\prime }p_{4}+p_{6}^{\prime }p_{8}+p_{7}^{\prime }p_{12}\right)\\ s=\frac{\left(p_{9}^{\prime }p_{4}+p_{10}^{\prime }p_{8}+p_{11}^{\prime }p_{12}\right)}{\left(p_{9}^{\prime }u+p_{10}^{\prime }v+p_{11}^{\prime }\right)} \end{array},$$

where $\left(x_{w},y_{w}\right)$ is the vehicle’s location in the world coordinates.

### 5.2. Vehicle Localization Performance

#### 5.2.1. Vehicle Localization in Static Condition

**Figure 6:**
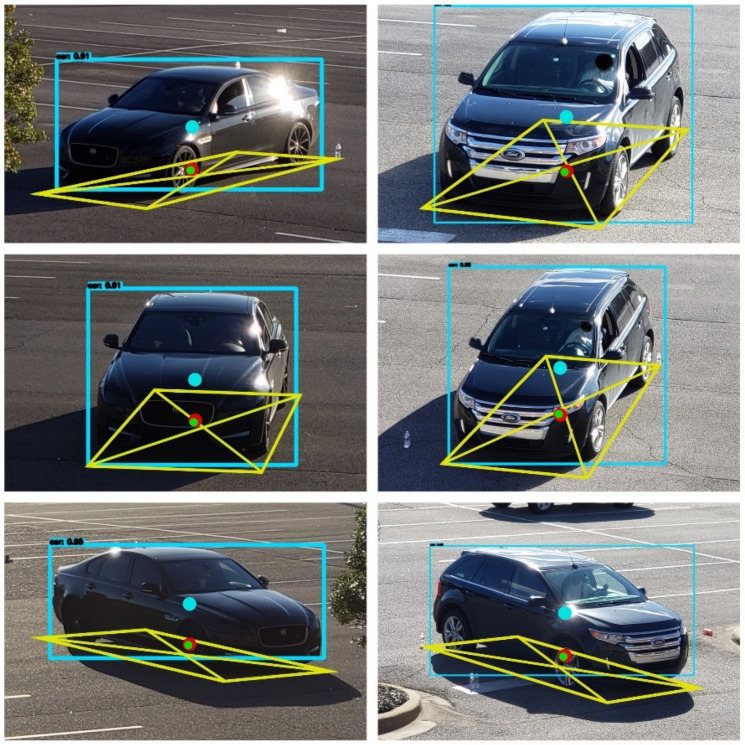
Corrected center positions of two vehicles with different orientations.

The performance of the vehicle center error method was quantitatively evaluated using two different types of vehicles with various orientations and distances as shown in Figure 6. To diversify the vehicle orientation in the images, three different horizontal locations (left, middle, and right), and two different heights (high and low) are used for the camera location. Additionally, the distance between the camera and the vehicles ranges from 30 to 80 m. A total of 24 still images are used to validate the proposed method in a static situation. In the figure, the centers of the vehicles obtained by bounding boxes are shown as blue dots, and the vehicle centers corrected by a linear regression model and a neural network are shown as red dots and green dots, respectively. The differences between the centers of the bounding boxes and the corrected vehicle centers can be clearly seen in the figure. However, since the vehicle localization error in the horizontal u-coordinate is negligibly small compared to that in the vertical v-coordinate in the image coordinates, the error in the u-coordinate is ignored and only the error in the v-coordinate is considered in this research.

The errors in localizing vehicle centers with and without error correction models are quantitatively measured and presented in Table 1. The true vehicle centers on the ground are determined by converting the intersection of the diagonals connecting the four corners of each vehicle into the 3D coordinates. The percent improvements are also calculated by comparing the errors before and after applying the regression models and shown in the parentheses. From the table, it can be seen that the neural network model shows slightly better results than the linear regression model mostly for the Sedan. On the contrary, the linear model shows slightly better results for the SUV. The slightly different results are deemed to be related to the different aspect ratios of the vehicles and the different learning capabilities of the regression models.

#### 5.2.2. Vehicle Localization in Dynamic Condition

**Figure 7:**
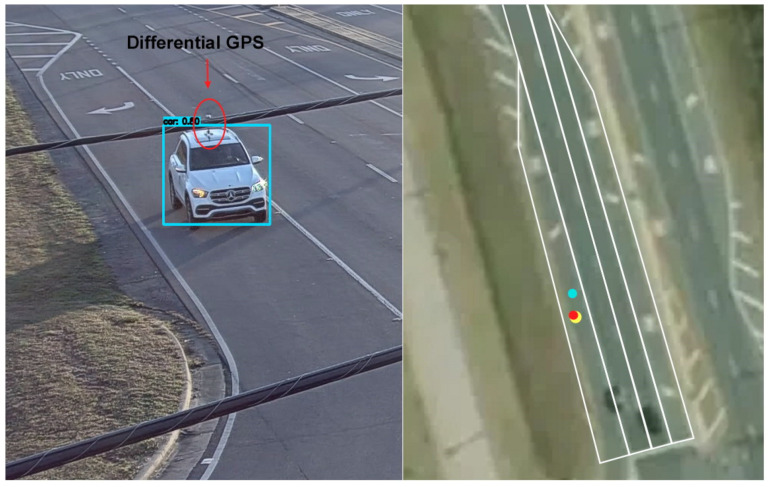
A vehicle equipped with a differential GPS receiver and its location in an aerial view map.

The performance of the vehicle localization method at a traffic intersection was quantitatively evaluated using a differential GPS as the reference. A vehicle equipped with a high-accuracy differential GPS receiver was driven through an intersection as shown in Figure 7. The figure depicts the moving vehicle, along with its location in the world coordinates. In the figure, the yellow dot represents the vehicle location measured by the differential GPS, the blue dot represents the center of bounding box without center correction, and the red dot represents the vehicle center corrected by a linear regression model. For the analysis, over one hundred data points were collected in each lane.

A comparison was made between the vehicle localization errors with respect to differential GPS measurement with and without vehicle center correction, and the results are shown in Table 2. The error was calculated by using the Euclidean distance, with the longitudinal error accounting for around 80–90% and lateral error accounting for just 10–20% in the world coordinates. Since the results obtained by the linear regression and neural network models are very similar, only the results obtained by the linear regression model are presented in the table. In the table, it is clearly seen that the errors corrected by the linear regression model are much smaller than those without any correction with an average improvement of 71.32%.

This result is better than the previously reported results by Lu et al. [21], which was described in Section 2. Their results showed that the average localization error was 1.81 m using a differential GPS as a reference and 1.68 m using a drone as a reference. In contrast to their approach where multiple cameras were used to generate 3D bounding boxes, the new approach presented in this paper achieved more accurate vehicle localization using only a single camera system and 2D bounding boxes. As a result, our method allows for faster processing speeds and greater efficiency.

### 5.3. Vehicle Counting Performance

**Figure 8:**
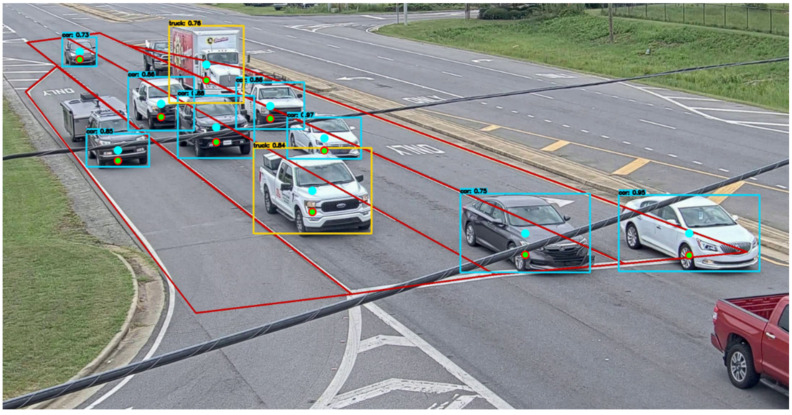
Vehicle detection and localization in real-time video captured by a traffic camera.

The proposed vehicle center error correction method was applied to a real-time traffic video captured by a camera as shown in Figure 8. The video camera was installed on a traffic signal pole at an intersection where the speed limit is 55 mph. The area of the camera image where vehicles are detected was pre-defined to only include the lanes that lead toward the intersection. This was performed to ensure that only vehicles moving in the direction of the camera are detected and taken into consideration. In the figure, the blue, red, and green dots within each bounding box represent the center of the bounding box, corrected center by the linear regression model, and corrected center by the neural network model, respectively. Due to the proximity of the vehicle centers corrected by the linear regression and the neural network models, the distance between those centers in the image coordinates is only a few pixels, making the red and green dots look almost identical.

**Figure 9:**
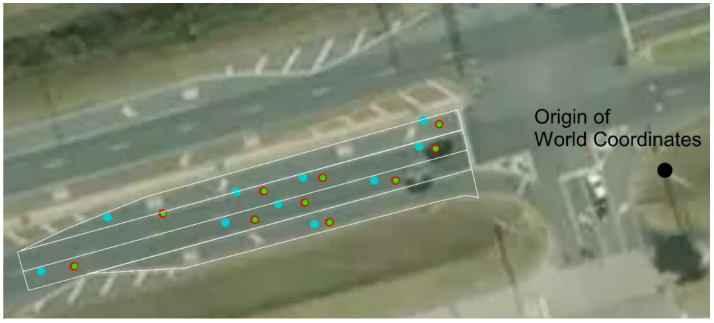
Vehicle locations in the world coordinates after coordinate transformation.

Using the corrected center locations of the vehicles in the image coordinates as input, a more accurate location of the vehicles in the 3D world coordinates can be obtained by the coordinate transformation. Therefore, obtained vehicle centers are plotted along with the corresponding centers of bounding boxes in the 3D world coordinates, as shown in Figure 9. In the figure, it can be seen that significant improvements have been achieved in the determination of the vehicle locations in the world coordinates, which allows a more accurate count of vehicles in each lane. Based on the accurate location measurement, it would also be possible to calculate the distance between vehicles and the speed of each vehicle if necessary. As seen from the single vehicle cases, the results from the linear regression and the neural network models are very close, and thus the red and green dots appear almost identical.

To quantitatively evaluate the accuracy of the proposed approach in vehicle counting for each lane, the vehicle localization error correction models were applied to two video clips captured by the traffic camera in two different traffic conditions: light and heavy. Each video was one minute long and captured at the rate of 30 frames per second, which generates a total of 1800 still images. Using a 2080Ti NVIDIA graphics card, a Python code combining YOLO and the error correction algorithms could process high-resolution images of 3072 × 1728 pixels at the rate of 30 frames per second in real time. To calculate the accuracy, all 1800 still images were manually checked to count the number of vehicles present in each lane, which was used as the ground truth. The accuracies in the vehicle count with and without the vehicle localization error correction method are shown in Table 3.

When no error correction method was applied, the accuracy in the vehicle count was the lowest for the left lane and gradually increased toward the right lane. The accuracy for the left lane is especially low compared to other lanes. This is due to the large azimuth angle, θ, and the elevation angle, 𝜙, for the vehicles in the left lane. As a result, a good portion of the vehicles in the left lane are assigned out of the lane resulting in a very low accuracy. This trend improves toward the right lane as the view angles change favorably. Additionally, the overall accuracy with the error correction method was slightly lower for the heavy traffic scenario than for the light traffic scenario. This is due to the fact that more vehicles are obscured by other vehicles in front, avoiding detection in the heavy traffic condition. With the error correction method, the overall accuracy improved significantly. Since the results obtained with the two different regression models did not show much difference, their results are combined and represented by a single value for each case. The accuracy of the proposed approach ranging from 73.95% to 98.35% over the two different scenarios is comparable to the accuracy ranging from 70.58% to 99.63% obtained by a previously reported method in [20]. However, the simplicity of the proposed approach provides added value when compared to the existing complex system.

## 6. Conclusions

In this paper, a simple and effective approach was presented to compensate for an error in localizing a vehicle center detected by a single traffic camera and processed by an object detection algorithm called YOLO. The error between the center of a vehicle’s bounding box and the real center of the vehicle projected on the road in image coordinates was corrected by using two different types of regression models: a linear regression model and a neural network. The models were trained with rectangular prism images generated at different distances and view angles, and the calculated center error was used as the output of the models. When tested with stationary vehicles in a parking lot and a moving vehicle at a traffic intersection, the results showed that the overall errors were reduced by around 85% and 71%, respectively. This approach was also applied to a live-streamed traffic video to quantitatively evaluate the accuracy of the proposed method in counting the number of vehicles in each approaching lane. The error correction method improved the accuracy of vehicle counting, with results ranging from 73% to 98%. In contrast, the basic vehicle detection algorithm YOLO had lower accuracies, ranging from 5% to 94%. The presented approach can be used to determine accurate traffic conditions at traffic intersections so that an adaptive traffic control system can make an optimal decision based on the correct number of vehicles in each approaching lane.

In this research, all tests were conducted on sunny days with adequate illumination. However, poor weather or low light conditions can negatively impact the accuracy of the object detection algorithm, YOLO, resulting in incorrect bounding box placement for vehicle detection. To improve vehicle detection performance under such conditions, additional sensors such as radar or LiDAR can be incorporated, and a sensor fusion algorithm can be applied. These enhancements can improve vehicle detection and minimize the negative impact of low illumination on the proposed vehicle localization method.

## Figures and Tables

**Table 1:** Comparison of vehicle center error corrections by linear and nonlinear regression models (better results shown in bold).

Vehicle Type and View Direction	Error without Correction (m)	Error Corrected by Linear Regression Model (m)	Error Corrected by Neural Network Model (m)
Sedan from right	3.49	0.58 (83.19%)	**0.52 (84.96%)**
Sedan from middle	2.94	0.52 (82.14%)	**0.36 (87.50%)**
Sedan from left	3.77	0.94 (75.00%)	**0.84 (77.59%)**
SUV from right	3.50	**0.16 (95.36%)**	0.21 (93.78%)
SUV from middle	1.76	**0.10 (94.16%)**	0.13 (92.36%)
SUV from left	2.48	0.59 (76.13%)	**0.56 (77.10%)**
**Average**	2.99	0.47 (84.33%)	**0.43 (85.55%)**

**Table 2:** Comparison of vehicle localization error with respect to differential GPS measurement in the world coordinates with and without center correction by a linear regression model.

Lane	Error without Correction (m)		Error Corrected by Linear Regression Model (m)		Number of Data Points
	Average	Variance	Average (% Improvement)	Variance	
Right	3.69	0.41	0.85 (76.96%)	0.14	124
Middle 1	5.54	1.87	0.77 (86.10%)	0.20	147
Middle 2	5.69	2.11	1.91 (66.43%)	0.89	109
Left	6.26	1.93	2.55 (59.27%)	0.90	125
**Average**	5.30	1.58	1.52 (71.32%)	0.51	126

**Table 3:** Vehicle count and accuracy for different lanes in light and heavy traffic scenarios. NC represents YOLO results without vehicle localization error correction, CC means YOLO results with the vehicle localization error correction, and GT is the ground truth.

	Light Traffic Scenario					Heavy Traffic Scenario				
	The Number of Vehicles			Accuracy (%)		The Number of Vehicles			Accuracy (%)	
	NC	EC	GT	NC	EC	NC	EC	GT	NC	EC
Left lane	18	211	246	7.32	85.77	17	298	403	5.70	73.95
Middle 1 lane	773	1236	1290	59.92	95.81	792	1885	1976	40.10	95.39
Middle 2 lane	1221	1302	1340	91.11	97.16	1862	2024	2107	88.37	96.06
Right lane	172	179	182	94.51	98.35	274	285	291	94.16	97.94

## Data Availability

Data sharing not applicable.
